# Role of antiviral therapy in reducing recurrence and improving survival in hepatitis B virus-associated hepatocellular carcinoma following curative resection (Review)

**DOI:** 10.3892/ol.2014.2727

**Published:** 2014-11-21

**Authors:** CHAOHUI ZUO, MAN XIA, QUNFENG WU, HAIZHEN ZHU, JINGSHI LIU, CHEN LIU

**Affiliations:** 1 Department of Gastroduodenal and Pancreatic Surgery, Translation Medicine Research Center of Liver Cancer, Hunan Province Tumor Hospital and Affiliated Tumor Hospital of Xiangya Medical School, Central South University, Changsha, Hunan 410013, P.R. China; 2 Department of Pathology, Immunology and Laboratory Medicine and Shands Cancer Center, University of Florida, Gainesville, FL 32610-0275, USA; 3 Department of Gynaecological Oncology, Hunan Province Tumor Hospital and Affiliated Tumor Hospital of Xiangya Medical School, Central South University, Changsha, Hunan 410013, P.R. China

**Keywords:** hepatocellular carcinoma, hepatitis B virus, recurrence, interferon-α, survival rates, curative resection, sorafenib, transatheter arterial chemoembolization

## Abstract

Hepatocellular carcinoma (HCC) is one of the major causes of cancer-related mortality worldwide, with the majority of cases associated with persistent hepatitis B virus (HBV) or hepatitis C virus infection. In particular, chronic HBV infection is a predominant risk factor for the development of HCC in Asian and African populations. Hepatic resection, liver transplantion and radiofrequency ablation are increasingly used for the curative treatment of HCC, however, the survival rate of HCC patients who have undergone curative resection remains unsatisfactory due to the high recurrence rate. HCC is a complex disease that is typically resistant to the most commonly used types of chemotherapy and radiotherapy; therefore, the development of novel treatment strategies is required to improve the survival rate of this disease. A high viral load of HBV DNA is the most important correctable risk factor for HCC recurrence, for example nucleos(t)ide analogs improve the outcome following curative resection of HBV-associated HCC, and interferon-α exhibits antitumor activity against various types of cancer via direct inhibitory effects on tumor cells, anti-angiogenesis, enhanced immunogenicity of tumors, immunomodulatory effects and liver dysfunction. In the present review, antiviral treatment for HBV-associated HCC is described as a strategy to reduce recurrence and improve survival.

## 1. Introduction

In males, liver cancer is the fifth most frequently diagnosed type of cancer and the second most common cause of cancer-related mortality, worldwide. However, in females, liver cancer is the seventh most frequently diagnosed type of cancer and the sixth most common cause of cancer-related mortality worldwide. . An estimated 748,300 new cases of liver cancer and 695,900 cancer-related mortalities occurred worldwide in 2008 (1), half of which were estimated to have occurred in China (2,3). The highest liver cancer rates occur in East and Southeast Asia, and in Central and Western Africa, whereas the lowest rates occur in South-Central and Western Asia, as well as in Northern and Eastern Europe. Globally, rates are more than twice as high in males as in females. Among the various types of primary liver cancer, hepatocellular carcinoma (HCC) is the predominant histological subtype, with chronic hepatitis B virus (HBV) and hepatitis C virus (HCV) infection attributable to ~75–80% of HCC cases worldwide (4,5). In particular, chronic HBV infection is a predominant risk factor for HCC development in Asian and African populations (6,7). HCC is a rare type of cancer in terms of its continued increase in incidence over a number of years (8,9). Additionally, HCC is the most relevant example of virus and inflammation-associated cancer; HBV and HCV are etiological factors for HCC, with persistent active hepatitis and hepatic fibrosis important in the development of HCC. The prognosis of HCC associated with hepatitis virus infection remains poor due to the high rate of HCC recurrence following surgery (10,11) and progressive liver dysfunction that results in hepatic failure. Hepatic resection, liver transplantation and radiofrequency ablation (RFA) are increasingly used for the curative treatment of HCC, and are able to achieve good local control of the disease (12). Liver transplantation is the most radical treatment for HCC, as well as for underlying liver diseases, such as chronic HBV or HCV infection (13,14); however, a shortage of donors hinders the efficacy of liver transplantation (15). In patients with small HCC, percutaneous RFA demonstrated similar local control and long-term survival rates to hepatectomy and, notably, percutaneous RFA is associated with a lower complication rate and shorter hospital stay (16). Although percutaneous RFA may provide small HCC patients with therapeutic effects similar to those of hepatic resection, it is more likely to result in the incomplete treatment of small HCCs located at specific sites in the liver; therefore, an open surgical approach to hepatic resection may be a preferable treatment strategy (17). Furthermore, RFA appears to be a reasonable strategy for the treatment of very small HCCs (<2 cm) with no technical contraindications, in which complete necrosis is likely to occur. In larger nodules (>2 cm and particularly >3 cm) and/or in tumor locations in which RFA is not expected to be effective or safe, surgical resection is preferred (18).

Surgery remains the most effective HCC treatment strategy with curative potential; liver resection is the gold standard for patients with resectable HCC that develops in otherwise healthy patients (19,20) and offers an acceptable outcome for carefully selected HCC patients (21,22). Refinements of surgical techniques and staging systems have resulted in five-year survival rates of ~50–70% following surgical resection (23,24). The application of advanced surgical techniques and instrumentation decreases blood loss, increases positive outcomes, such as survival and quality of life, and decreases the requirement for the Pringle maneuver compared with portal triad clamping (25–28). Although liver resection appears to be the most effective strategy for the treatment of HCC, patients who undergo curative resection often exhibit a high rate of relapse (29,30) and only ~10–20% of patients with HCC are currently eligible for surgical intervention. Considering its apparent superiority to liver transplantation and local ablative therapy, surgical resection is likely to remain the primary approach for patients who present with very early- and early-stage HCC. Furthermore, the risk factors of tumor size, tumor nodules, vascular invasion, chronic viral infection, liver function and portal pressure have been identified as prognostic predictors of recurrence following liver resection (31–33). HCC development is a multistep process that typically occurs over a number of years; numerous mutations accumulate in the cellular DNA, resulting in malignant transformation, growth and metastatic behavior of the cell. Recently, it has become apparent that the tumor microenvironment, as well as the tumor cell itself, are key in the development of the tumor. For example, in the liver cancer microenvironment, a direct association appears to exist between the role of inflammation and the development of cirrhosis (34–36). Multicentric recurrence of HCC is closely associated with persistent active hepatitis and hepatic fibrosis, and costimulation affects intrahepatic immune responses, appearing to be important in immune tolerance, immune injury and immune abnormalities in patients exhibiting chronic HBV infection (37–39). Thus, the present review highlights the mechanism of HBV induction of HCC and discusses the current trends in antiviral therapy following curative resection of HCC.

## 2. HBV and the risk of HCC recurrence

HCC will almost invariably occur in a histologically abnormal liver, with the existence of chronic liver disease representing a potential risk for the development of this type of tumor. In addition to the specific viral mechanisms that may be directly carcinogenic to the liver, chronic necroinflammation and the accumulation of reactive oxygen species contribute to carcinogenesis via chromosomal injury (40). Cirrhosis, the end-stage consequence of hepatic inflammation that results in nodular transformation of the liver, is considered to be a premalignant condition, independent of its etiology.

Numerous studies have attempted to summarize the pathogenesis of HBV-associated carcinogenesis, as well as the viral and host factors that may increase the risk of developing HCC. HBV is the predominant cause of chronic hepatitis, cirrhosis and HCC (41–43); a high viral load of HBV is crucial for the development of chronic liver disease and a previous study using long term tracking of HBV viral load determined the association between HBV viral load and the risk of developing HCC (44). Furthermore, epidemiological studies have examined differences in HCC carcinogenesis due to HBV DNA load (45), however, the mechanisms by which HBV DNA load causes differences in malignant transformation remains unclear. It has recently been demonstrated that high HBV load is associated with an increased risk of developing HCC in patients with chronic HBV (46), and HBV DNA load has recently been found to be associated with HCC recurrence following curative treatment (47).

Sohn *et al* (48) investigated the predictive role of HBV DNA and HBV surface antigen (HBsAg) levels in the early and late recurrence of HCC following curative resection in patients with HBV-associated HCC. A total of 248 patients underwent curative resection for HBV-associated early-stage HCC (group one, solitary tumor; group two, <5-cm diameter or multinodular tumor; group three, ≤3 tumors and <3-cm diameter). Multivariate analysis identified risk factors for early recurrence, including the presence of microvascular invasion [hazard ratio (HR), 3.86; P<0.001], preoperative HBV DNA levels of ≥ 20,000 IU/ml (HR, 2.77; P<0.001) and des-γ-carboxy prothrombin levels of ≥40 mAU/ml (HR, 1.76; P=0.045). HBV DNA levels were associated with early recurrence, whereas HBsAg levels were associated with late recurrence following curative resection for HBV-associated HCC. Additionally, Xia *et al* (49) reported that high serum hyaluronic acid levels and high HBV viral load were the principle prognostic factors associated with local recurrence following complete radiofrequency ablation for HBV-associated small HCC. As HBV DNA load changes with the administration of antiviral agents, patients with a high viral load at the time of HCC treatment who receive antiviral agents subsequently demonstrated differences in HBV DNA load compared with those who did not receive antiviral agent therapy. An *et al* (50) examined 188 patients with HBV-associated HCC who underwent curative resection and performed multivariate analysis to identify the following independent predictors of HCC recurrence in all participants: A tumor size of >5 cm, Child-Pugh class B, vascular invasion and >10^4^ copies/ml HBV DNA at resection. This high HBV replication state was the chief predictor of a poor outcome following resection of HBV-associated HCC. By contrast, multivariate analysis revealed that a sustained suppression of <10^4^ copies/ml HBV DNA was the sole factor that resulted in low HCC recurrence and an extended survival period, acting as a strong protective factor for long-term recurrence-free and overall survival . During HBV-induced HCC initiation, chronic inflammation typically facilitates the evolution of HBV mutants which promote HCC development. Therefore, the development of efficient preventative and therapeutic strategies against HCC in HBV-infected patients may rely on investigations into the effects of inflammation on HBV-induced HCC initiation and progression (51–54).

## 3. Mechanism of IFN-α in HBV-associated HCC

Interferon-α (IFN-α) belongs to the type I IFN family of cytokines, which were originally identified and isolated for their antiviral properties (55–57). Subsequent studies revealed that IFN-α exhibits antitumor activity against various types of cancer by directly inhibiting tumor cells; this includes preventing angiogenesis, enhancing the immunogenicity of tumors and performing immunomodulatory effects on tumor cells (58,59). Chronic HBV infection has been treated with IFN-α for >20 years and IFN-α was one of the first agents approved by the Food and Drug Administration (FDA) for this purpose (60). IFN-α induces the expression of various antiviral host proteins, such as protein kinase R and myeloid differentiation primary response protein 88. To exert its antitumor activity, IFN-α may accelerate tumor necrosis factor-induced tumor cell apoptosis by upregulating Fas gene expression (61); however, opposing studies have demonstrated that pretreatment with IFN-α inhibits tumor necrosis factor-related apoptosis-inducing ligand (TRAIL)-mediated nuclear factor-κB (NF-κB) activation, thereby increasing the response of hepatoma cells to the TRAIL-induced apoptosis signal (62). Thus, for patients with HBeAg-positive chronic HBV, percutaneous endoscopic gastrostomy (PEG) combined with IFN-α offers superior efficacy on the basis of HBeAg and HBsAg seroconversion, as well as HBV DNA suppression. As a result of these features, novel therapeutic regimens based on combinations of PEG IFN-α with third-generation nucleos(t)ide analogs (NUCs), such as entecavir and tenofovir, are being developed to increase the rate of HBsAg seroclearance, which remains the ideal endpoint for all HBeAg-negative chronic HBV patients.

Liu *et al* (63) determined that IFN-α may improve chemosensitivity in tumor cells by inhibiting HBV X protein (HBx)-induced activation of the NF-κB signaling pathway. Additionally, it was proposed that IFN-α may aid in reverting previously acquired chemoresistance. Consistent with the findings that IFN-α inhibits HBx-mediated activation of NF-κB, Liu *et al* concluded that HBx-induced resistance to therapeutic agents was associated with the activation of the NF-κB signaling pathway. These results indicate that IFN-α may be a useful adjuvant treatment to chemotherapy for enhancing the response of HBV-associated HCC via HBx-triggered NF-κB activation. The mechanisms involved in the process whereby HCC establishes an immunologically tolerant tumor environment remain poorly characterized. Cabrera *et al* (64) showed that HCC patients have blunted T cell immunity that is partly associated with elevated levels of soluble interleukin-2 receptor α chains, supporting a novel immuno-inhibitory role for this soluble receptor. In HepG2 and HepG2.2.15 cells, Hou *et al* (65) revealed a novel role of IFN-α and microRNA (miR) 146a in HBV immunopathogenesis, and provided a potential target for the therapeutic recovery of anti-HBV effects.

Conclusive studies in relevant experimental systems have yet to be conducted to determine the molecular mechanisms by which IFN-α suppresses HBV replication, however, it is known that the HBV genome contains an IFN-stimulated response element (IRSE) (66). Belloni *et al* (67) identified that IFN-α decreases the transcription rate of pregenomic (pgRNA) and subgenomic RNA in the covalently closed circular DNA (cccDNA) minichromosome of HBV, inhibiting HBV replication. However, this experiment was only conducted in cultured cells in which HBV was undergoing replication, and in mice whose livers had been repopulated with human hepatocytes and infected with HBV. The administration of IFN-α in these two systems resulted in transcriptional inhibition by multiple mechanisms: cccDNA-bound histones were hypoacetylated, transcriptional corepressors were actively recruited to the cccDNA, and binding of signal transducer and activator of transcription 1 (STAT1) and STAT2 to active cccDNA was reduced. IRSE-mutant HBV exhibited a reduction in pgRNA transcription and was resistant to IFN-α-induced repression; therefore, the abovementioned inhibitory effects of IFN-α were associated with IRSE via epigenetically-mediated HBV cccDNA transcriptional repression. Additional understanding of this molecular mechanism may assist in the development of effective novel therapeutic strategies.

In order to conduct more relevant experiments regarding the association between IFN-α and hepatitis infection, Yang *et al* (68) established a hepatoma cell line; cells were isolated from the liver tumor tissue of a male patient with chronic HCV infection (Hunan Provincial Tumor Hospital, Changsha, China) to create the HLCZ01 cell lines, which was the first cell line to support the entire life cycle of HBV and HCV. The ability of IFN-α to inhibit HBV replication in HBV-infected HLCZ01 cells was examined and it was identified that IFN-α inhibits HBV replication in the culture system by decreasing the transcription of viral pregenomic RNA. In a systematic review of 11 studies of the effect of IFN-α and NUC therapy on the outcome of HBV infection over the previous 10 years, Sung *et al* (69) determined that IFN-α or NUC treatment significantly reduced the risk of developing HCC. Although IFN-α therapy benefited patients with cirrhosis, NUCs benefited those with non-cirrhosis and HBeAg-positive infections. Considering the abovementioned studies, sustained HBV suppression induced by IFN-α and NUC therapy may be necessary to reduce the development of HCC in HBV-infected patients.

Thus, the following mechanisms contribute to the antitumor effect of IFN: First, IFN may cause the induction of pro-apoptotic genes; second, IFN may directly effect malignant cells; third, IFN may inhibit angiogenesis; and last, it may augment antitumor immune responses and improve liver function.

## 4. Role of IFN-α on HBV-associated HCC recurrence following surgery

Although curative resection represents the preferred method for extending the survival of HCC patients, the survival rate remains poor due to the high rate of HCC recurrence (70–72). A systematic review of thirteen randomized control trials indicated that the beneficial effects of adjuvant IFN-α therapy reduced the rate of recurrence and IFN-α did not appear to improve the survival of HCV-associated HCC patients following curative therapy (73). However, in a retrospective cohort study conducted by Qu *et al* (74), 568 HBV-associated HCC patients who underwent curative resection were investigated; Postoperative IFN-α therapy (5×10^6^ U IFN-α; 3 doses/week) was received by 101 patients for 18 months and clinicopathological factors were compared between patients who did or did not receive postoperative IFN-α therapy. Patients who received postoperative IFN-α therapy exhibited higher overall survival rates [HR, 0.612; 95% confidence interval (CI), 0.422–0.889; P=0.010), however, no significant difference was identified in the disease-free survival rates between the two groups (HR, 0.786; 95% CI, 0.597–1.035; P=0.086). Additionally, multivariate analysis revealed that postoperative IFN-α therapy was an independent factor for significant reductions in overall survival rates (HR, 0.611; 95% CI, 0.421–0.887; P=0.010) and early recurrence rates (HR, 0.562; 95% CI, 0.375–0.840; P=0.005). This previous study demonstrated that IFN-α therapy for patients exhibiting HBV-associated HCC following curative resection prevents early recurrence rates and improves overall survival. In addition, a different retrospective study identified that patients with a persistent level of ≥4log_10_ copies/ml HBV DNA upon resection and follow-up demonstrated the highest risk of recurrence (HR, 4.129; P<0.001); and ≥4log_10_ copies/ml HBV DNA upon resection was the most significant risk factor for HCC recurrence. Furthermore, the risk of recurrence was significantly reduced in patients who underwent postoperative IFN-α treatment following resection (75). In a randomized clinical trial (n=235), Sun *et al* (76) compared treatment (3 doses/week of 5×10^6^ U IFN-α intramuscularly for 18 months) and control groups following resection. Statistical analysis was based on the method of intent-to-treat and determined that IFN-α treatment improved the overall survival of patients with HBV-associated HCC following curative resection, possibly by postponing recurrence. Additionally, in 27 randomized controlled trials predominantly conducted in Asian populations, comparing adjuvant with no adjuvant therapy, Wang *et al* (77) reported that adjuvant chemotherapy, internal radiation and heparanase inhibitor PI-88 therapy failed to improve recurrence-free survival or overall survival rates, however, adjuvant IFN-α therapy did improve recurrence-free and overall survival rates. Furthermore, the combination of systemic and transhepatic arterial chemotherapy was not recommended for HCC following potentially curative treatment, therefore, postoperative IFN-α therapy may be beneficial for HBV-associated HCC patients (78,79).

The benefits of adjuvant IFN-α therapy for patients who have undergone resectable HCC are controversial. In a total of 268 Tawainese patients, a recent phase III randomized study of IFN-α2b therapy following curative resection for HBV- and HCV-associated HCC demonstrated no preventative effect on HBV or HCV recurrence (80). Therefore, the role of IFN-α in improving survival may be associated with different patients responses (81). miRNAs can be used as biomarkers for diverse types of malignancies. Notably, miRNAs can be utilized to investigate various cancer phenotypes, used as therapeutic agent targets or used as the therapeutic agent itself (82–84). IFN-α may prolong the survival period in specific patients, however, the response is often unsatisfactory as the identification of suitable patients who are likely to benefit from this therapy is difficult. Therefore, the development of molecular tools for the classification of patients with respect to their response to IFN-α therapy is required. In 214 patients from two independent, prospective, randomized, controlled trials of adjuvant IFN therapy, Ji *et al* (85) employed quantitative reverse transcriptase-polymerase chain reaction to determine miRNA expression levels and assess their association with survival rates and response to IFN-α therapy. This analysis identified that the expression patterns of miRNAs in liver tissue differ between male and female HCC patients, and that the miRNA-26 expression status of such patients is associated with survival and response to IFN-α adjuvant therapy. Furthermore, Ji *et al* (86) developed a miR26-diagnostic test, which may assist in the selection of candidate HCC patients who exhibit a favorable overall survival response to adjuvant IFN-α treatment. Such a test may prevent unnecessary IFN-α treatment for individuals who do not exhibit improved survival, thereby providing them with an opportunity to undergo alternative treatment modalities. Hou *et al* (87) identified that the expression of IFN-stimulated retinoic acid inducible gene-I (RIG-I) was significantly downregulated in human HCC tissues. Additionally, a lower RIG-I expression level was associated with a shorter survival period and a poorer response to IFN-α therapy, indicating that RIG-I is a useful prognostic factor and IFN-α response predictor for HCC patients. RIG-I appears to enhance the IFN-α response by amplifying the activation of the STAT1 transcription factor*,* which in turn amplifies IFN-α effector signaling. Furthermore, this previous study identified that RIG-I deficiency promotes the initiation of HCC development and determined that hepatic RIG-I expression levels are lower in male compared with female individuals. Thus, the RIG-I gene may act as a tumor suppressor in HCC and contribute to HCC gender disparity. Similarly to the miR26-diagnostic test, the implementation of a RIG-I test may prevent unnecessary IFN-α treatment for individuals who do not exhibit improved survival from this type of therapy, thereby, providing them with an opportunity to undergo alternative treatment modalities.

## 5. Synergistic effects of IFN-α and other agents on HBV-associated HCC recurrence following surgery

Transcatheter arterial chemoembolization (TACE) is a therapeutic treatment for HCC patients, which particularly implements its therapeutic effect in patients exhibiting moderate-to-advanced grade, inoperable HCC (88–90). However, the benefits of adjuvant TACE for patients with resectable HCC are controversial, for example, a number of clinical trials have identified no evidence supporting the benefits of adjuvant TACE for non-high-risk patients (91,92). Furthermore, Breunig *et al* (93) determined that the administration of adjuvant TACE produced a significant survival benefit in patients with risk factors of recurrence. For patients with large HCC tumors with multiple intrahepatic metastases, debulking surgery followed by IFN-α and 5-FU combination therapy offers the possibility of long-term survival; however, optimal debulking is essential for prolonged survival during subsequent treatment (94), for example, postoperative adjuvant TACE is typically administered to HCC patients at a high risk of recurrence (95,96). In 27 patients with large HCC tumors and multiple intrahepatic metastases, Tanaka *et al* (97) performed IFN-α and TACE combination therapy following maximal liver tumor resection, and identified that this treatment strategy offered the possibility of long-term survival despite the late stage of the disease. In a preliminary report of 33 patients, Kumamoto *et al* (98) proposed that combination of subcutaneous IFN-α and intra-arterial 5-FU infusion therapy may delay the development of recurrence and reduce the number of recurrent nodules in the remnant liver following curative resection. As a result, combination therapy may improve the prognosis of advanced HCC patients with portal vein invasion or intrahepatic metastasis. The prognosis of HBV-associated HCC is principally influenced by the risk of recurrence following curative resection, thus, the prevention and treatment of recurrent tumors may be required to improve the long-term survival rates of HCC patients. In a retrospective study (n=120), Yan *et al* (99) reported that postoperative administration of TACE prevents early HCC recurrence and the administration of antiviral therapy prevents the late recurrence of HCC. Therefore, antiviral and TACE combination therapy is proposed for the treatment of HCC patients with a high risk of recurrence following liver resection.

Sorafenib (BAY 43-9006; Nexavar; Bayer Pharmaceuticals Corp., West Haven, CT, USA) is a multikinase inhibitor, which can be orally administered. Sorafenib inhibits tumor cell proliferation by targeting the Raf/mitogen-activated protein kinase/extracellular signal-regulated kinase (Raf/MEK/ERK) signaling pathway and exerts an antiangiogenic effect by targeting a number of tyrosine kinases: Vascular endothelial growth factor-2 (VEGFR-2), VEGFR-3 and platelet-derived growth factor receptor-β (100,101). In preclinical models, sorafenib demonstrated dose-dependent activity against a wide range of tumor types, including HCC (102); it inhibited cell growth, induced apoptosis and downregulated the anti-apoptotic protein Mcl-1 via a Raf/MEK/ERK-independent mechanism. Sorafenib is approved by the US FDA for the treatment of HCC, based on phase two and three clinical trial data from patients with advanced metastatic HCC; the sorafenib treatment group demonstrated close to a three-month survival advantage over the untreated group (103,104). Furthermore, liver-directed therapy exhibited a response rate of >70%, and sorafenib has previously produced reasonable toxicity profiles and a slight improvement in therapeutic efficacy when administered in combination with liver-directed therapies (105); therefore, sorafenib must be considered as a viable treatment strategy for HCC patients, within the context of all currently available treatment options In a pilot study of 31 patients, Wang *et al* (106) performed a Cox regression analysis and identified that the administration of sorafenib was the only prognostic variable associated with HCC recurrence (HR, 0.24; 95% CI, 0.08–0.75; P=0.014). This previous study indicated that sorafenib may be an adjuvant therapy for HCC to prevent early recurrence following hepatic resection. Furthermore, the cumulative recurrence-free survival rate demonstrated the preventive effectiveness of sorafenib; sorafenib provides a significant survival benefit for prevention in HCC patients with a high risk of recurrence (107,108).

Kusano *et al* (109) demonstrated a synergistic antiproliferative effect of combination therapy on HAK-1B cells *in vitro*. Additionally, a significant reduction in tumor volume and weight was observed *in vivo* HAK-1B and KIM1 tumor cells administered with combination therapy, although synergistic effects were not obvious. The density of cluster of differentiation 34-positive microvessels was significantly lower and cleaved caspase-3-positive apoptotic cell numbers were higher in the sorafenib group and the combination group compared with the control or PEG-IFN-α2b groups in HAK-1B and KIM-1 tumors. Furthermore, the Ki67 labeling index was significantly lower in the combination group compared with the control group in KIM-1 tumors. Thus, the results of the study conducted by Kusano *et al* (109) indicate that combination therapy may be a more effective strategy for the treatment of HCC cases with variable sensitivity to the antitumor effects of singular sorafenib or PEG-IFN-α2b therapy. Additionally, xenograft experiments conducted by Wang *et al* (110) identified that IFN-α and sorafenib combination therapy on exhibited an enhanced effect on tumor growth inhibition and apoptosis induction *in vivo,* providing rationale for the clinical application of IFN-α and sorafenib combination therapy in HCC treatment, which may be use in clinical practice in the near future.

## 6. Conclusion

Currently, patients exhibiting high HBV DNA levels at HCC onset demonstrate significantly higher HCC recurrence rates compared with patients exhibiting low HBV DNA levels (111,112). The efficacy of tertiary prevention of HCC with any agent, including chemotherapy, HBV therapy or IFN, has yet to be determined, and safe and effective chemotherapy for HCC-recurrence has yet to be established, however, tumor prevention appears to be most effective in patients with chronic HBV infection (113). Treatment with PEG-IFN may be suboptimal as it results in significant adverse effects and NUCs may induce resistance. It is important to achieve HBsAg loss and anti-HBsAg conversion in therapy, as these outcomes produce the most favorable outcome (114).

In conclusion, the effects of antiviral therapy on reducing recurrence and improving survival in HBV-associated HCC following curative resection were reviewed in the present study. IFN-α may increase survival in patients administered with IFN-α therapy by preventing HCC recurrence following liver resection. Notably, the present review determined that HBV replication should also be monitored, as sustained HBV activation or relapse is significantly associated with HCC development and recurrence. Individualized antiviral therapy is important by measuring miRNAs to prevent unnecessary IFN-α treatment for those individuals who do not experience improved survival. RIG-I expression is a useful prognostic marker and IFN-α-response predictor for HCC patients. The combination of IFN-α and TACE is only proposed for prevention in HCC patients with high risk of recurrence. Furthermore, sorafenib is a newly introduced therapeutic agent, which offers improved prevention for HCC patients with high risk of recurrence. Future studies should focus on the synergistic role IFN-α and sorafenib in the management of chronic HBV-associated HCC following curative resection, and HBsAg loss and anti-HBV conversion, the development of safe and affordable agents, as well as management strategies to improve sustained or maintained HBV suppression, should be the future aims for the management of chronic HBV-associated HCC.
